# The Reality Monitoring Deficit as a Common Neuropsychological Correlate of Schizophrenic and Affective Psychosis

**DOI:** 10.3390/bs3020244

**Published:** 2013-05-03

**Authors:** Daniele Radaelli, Francesco Benedetti, Roberto Cavallaro, Cristina Colombo, Enrico Smeraldi

**Affiliations:** 1 Department of Neuropsychiatric Sciences, Scientific Institute and University Vita-Salute San Raffaele, Milan, 20127, Italy; 2 C.E.R.M.A.C. (Centro di Eccellenza Risonanza Magnetica ad Alto Campo), University Vita-Salute San Raffaele, Milan, 20132, Italy

**Keywords:** reality monitoring, neuropsychology, psychosis, schizophrenia, affective disorder

## Abstract

For many decades, Neuropsychological functioning has been a key point in the study of psychotic disorders. The main aim of these studies is to give a description of the neurocognitive “profile” of schizophrenia, with only little attention being paid to the common and discriminating features of different psychotic disorders. Recent studies support the hypothesis that patients affected by psychiatric disorders with psychotic symptoms have specific abnormalities of reality testing of ongoing perception, which become evident with source monitoring task. Ninety-eight patients and 50 controls were studied. Patients were divided by diagnosis and previous history of psychotic features and were administered Source Monitoring Task to test reality testing of ongoing perception. Frequencies of correct and false attributions were recorded. To obtain measures of observer sensitivity and response biases, a signal detection analysis was performed. Aims: Studying neuropsychological correlate of psychosis in euthymic mood disordered patients and patients with schizophrenia with or without delusions. Results: Patients with psychotic features use more lax criteria in evaluating self-generated, but not perceived stimuli compared to patients without psychotic features. Conclusions: Our findings support the hypothesis of selective biases in reality monitoring as neuropsychological correlates of psychosis.

## 1. Introduction

There has been a little attempt to study the common and discriminating features of psychotic symptoms. Strongly identified with schizophrenia, psychotic symptoms have traditionally been investigated in schizophrenic populations. A recent shift has led to a strategic focus on other clinical groups on the basis of observations that psychotic experiences are common in several psychiatric and also nonpsychiatric populations. Transdiagnostic studies may shed some light on the mechanisms that are specific to Psychosis independently of other symptoms [1]. In recent years, mounting evidence supported the hypothesis that patients affected by psychiatric disorders with psychotic symptoms have specific abnormalities in reality testing of ongoing perception, which become evident with source monitoring tasks (see review in [2]). Source monitoring refers to the set of processes involved in the attribution of an origin to memories and beliefs [3,4,5,6,7]. The vast majority of these studies have focused on schizophrenia, and found source monitoring errors consistently involving attribution of self-generated item to outside sources [8,9,10,11]. Recent studies show a specific correlation between source monitoring deficits and psychotic symptoms such as disorganization [12,13], confabulation [14], hallucinations [15] and symptoms of alien control [10,16].

The importance of the qualitative characteristics of memories and of an intact judgment process are especially clear when considering specific phenomena such as delusions. Delusions are likely to involve imagined sensory information and involve a loss of control over thoughts that come automatically, or unbidden. Following this way of reasoning source monitoring errors may lead to a wrong identification of imagined events as perceived. Similar considerations can be made for reality-testing errors that produce hallucinations**. **Some authors specifically suggest that certain hallucination phenomena occurring in hypnosis result from the suspension of a reality “monitor” [2].

Anselmetti *et al.* [11] found that the source attribution of recognized self-generated items was significantly biased towards external sources in delusional patients in comparison to controls. These findings may represent the relation between cognitive impairment and psychotic symptoms: due to the inability to identify the self as the source of internally generated information, the patient experience**s** it as originated from an external source in the form of symptoms such as delusions. 

Patients affected by major depression were reported to have wide-ranging deficits in several cognitive functions (see reviews in [17,18,19,20]), with psychotic depressed patients showing worse performances than nonpsychotic ones in some studies, [21] but not in others [22] in the absence of disease-specific impairments; our group had previously reported source monitoring deficits in delusional depression [23]. These clinical data supported the hypothesis that, in reality, monitoring deficits are associated with psychosis in general and may play a role in the development of delusions: delusional people could use abnormally lax criteria for the evaluation of mental experiences, and make misattributions about the source of information which may lead to incorrectly identifying imagined events as perceived [2,24] or the opposite.

The aim of the present study is to investigate the presence of source monitoring deficits in the psychotic state by testing reality monitoring in euthimic mood disordered patients with or without previous episodes of major depression with delusional features and in patients with schizophrenia with or without delusional features.

## 2. Materials and Methods

### 2.1. Subjects

Ninety eight patients and 50 controls were studied. All patients were in charge at the research center for mood disorders and schizophrenia of San Raffaele Hospital in Milan.

Forty four patients with a DSM-IV [25] diagnosis of mood disorder (unipolar) were divided in two groups: 23 without a history of psychotic features and 21 with a history of psychotic features. Inclusion criteria were: a previous depressive episode with presence or absence of delusional symptoms observed by the psychiatrist in charge; stable euthymia (at least one year); stable long-term treatment (at least six months without changes in drug treatments); absence of concurrent Axis II disorder. 

Fifty four patients with DSM-IV [25] diagnosis of schizophrenia were recruited and divided in two subgroups: 19 non-delusional at the time of the test and 35 delusional patients. Exclusion criteria were: history of substance dependence or abuse, co-morbid diagnosis on Axis II, epilepsy or any other major neurological illness or perinatal trauma. All patients had to be treated with a stable dose of the same antipsychotic monotherapy for at least 6 months. 

Fifty control subjects (23 males and 27 females) without any Axis I or Axis II relevant psychopathology or history of psychotic disorders in first-degree relatives were recruited. 

### 2.2. Psychopatological Assessment

Psychopatological assessment included two measures of paranoid thinking: the Fenigstein and Vanable Paranoia Scale (FVPS) [26] and the Internal, Personal and Situational Attribution Questionnaire (IPSAQ) [27]. The IPSAQ is a 32 items scale that describe 16 positive and 16 negative social situations in the second person (e.g., “A friend betrays the trust you placed in them”). The respondent is required to generate causal explanations, categorized as internal (something to do with the respondent), personal (something to do with another person or persons), or situational (something to do with circumstances or chance). Three positive and three negative subscale scores are generated by summing the number of times internal, external-personal, or external-situational attributions are chosen for positive and for negative items. Two derivative scores are produced: Externalizing Bias (EB, number of internal attributions for positive events minus number of internal attributions for negative events) and Personalizing Bias (PB, proportion of external attributions for negative events made to personal loci). Previous studies showed that paranoid patients tended to choose external attributions that located blame in other individuals, with higher EB and PB scores, while depressed patients did the opposite [27]. Schizophrenic patients psychopathology was assessed by means of the Positive and negative Symptoms Scale for Schizophrenia (PANSS), administered by a trained psychiatrist; while depressive symptomatology was assessed with the Hamilton Rating Scale for Depression (HAM-D) [28].

### 2.3. Source Monitoring Task

The task is the same paradigm used by Keefe [10]. Participants were asked to remember word items. During the study phase these items were identified by the participant through word-stem completion (self-generated condition), pictures (picture condition) or read by the experimenter (heard condition). Word items (16 categories and 8 words within each of those categories) were selected from an Italian version of the Battig and Montague (1969) category norms [29]. Picture items were simple black and white pictures on 4x4 note card and were taken from the Picture Database Norms on Italian Population from Job, Lotto and Dell’Acqua (2000) [30]. Heard items were read by the experimenter and repeated by the subject. Self-generated items were printed on 4x4 note cards with two or three letters missing from each word (example, B_N_N_ for “banana”). All participants were given the same items in the same order (for every category, two self-generated items, two picture items and two heard items). They were instructed to remember the presented category items and their sources. After completing all categories the experimenter gave the participant a test sheet that listed the 48 words presented plus 16 new items as distractors in the same order for each participant. The subject was asked to check one of the four response options (saw a picture, read by the experimenter, generated by filling the blanks, not presented before). The test was self-paced.

### 2.4. Data Analysis

Frequencies of correct and false attributions were recorded. To obtain measures of observer sensitivity and response biases, a signal detection analysis was performed by means of a computerized program (RscorePlus) [31]. Measures of sensitivity (d') and response bias (β) were calculated for the three source conditions of the stimuli (self-generated, heard, pictures). The first measure, d', depends on both the individual’s inherent ability to discriminate between two alternatives and how effectively the material can be encoded into memory; in this study, d' was a measures of the subject’s ability to discriminate between perceived and imagined words. The second measure, β, reflects *a priori* subjective biases toward certain kind of decisions; depending on this criterion, patients could be more or less conservative in attributing the presented stimuli to a given source. Both measures have been successfully used in psychiatric neuropsychology, and could help in identifying specific biases in information processing [23,32,33,34]. The transformation logβ was used rather than β because it tends to produce a distribution close to normality, since statistical properties of β are still under evaluation [35]. Positive values of logβ indicate a strict criterion, whereas negative values indicate a lax criterion [33]. 

Clinical and demographic characteristics and test scores were compared with Student’s t-test.

Group analyses were made using an ANOVA statistic and post hoc were analyzed with Fisher LSD test.

## 3. Results

Clinical and demographic characteristics of the patients, and scores at FVPS and IPSAQ are resumed in Table 1. 

**Table 1:** Clinical and demographic characteristics of the patients and scores in the Fenigstein and Vanable Paranoia Scale (FVPS) and Internal, Personal and Situational Attribution Questionnaire (IPSAQ) questionnaires.

	Mood disorder		Schizophrenia		Controls		
	Delusional (n = 21)	Non delusional (n = 23)	Delusional (n = 35)	Non delusional (n = 19)	(n = 50)	F	*p*
**Sex (M/F)**	7/14	5/18	18/17	10/9	23/27		
**Age**	39.33	39.43	34.11	34.95	32.28	2.39	0.053
**Age at onset**	29.20	28.84	22.21	24.95		5.13	0.003
**FVPS score**	42.33	40.71	52.17	40.90	33.95	6.47	0.000
**IPSAQ Externalizing Bias**	3.00	2.57	3.08	4.36	5.30	1.94	0.111
**IPSAQ Personalizing Bias**	0.58	0.51	0.56	0.48	0.39	1.86	0.124

FVPS showed significant differences among groups, with post hoc analysis showing lower scores in controls than in both delusional depressed (*p* = 0.016) and schizophrenic (*p* < 0.0001) patients. 

In the IPSAQ questionnaire, pooled delusional schizophrenic and depressed patients showed lower tendencies to externalization and higher tendencies to personalization (EB: *p* = 0.032 and PB: *p* = 0.009) compared to controls.

**Figure 1:**
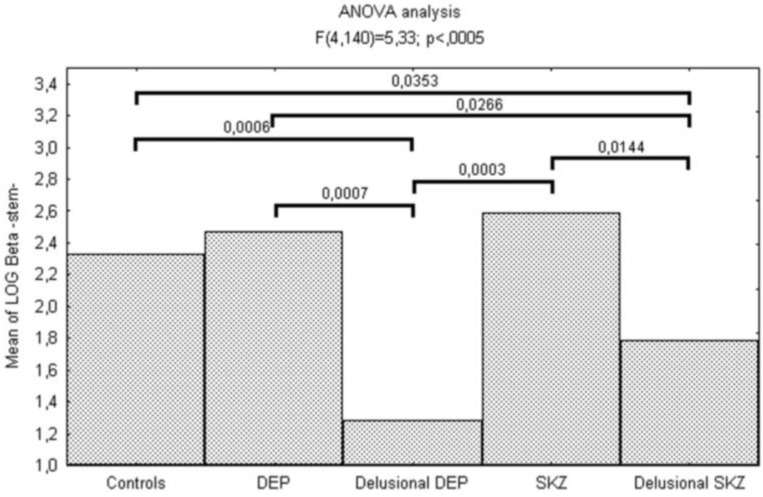
Reality monitoring deficits in schizophrenic and affective psychosis.

This bias toward personalization and against externalization in psychotic patients was confirmed by the signal detection analysis of the source monitoring task showing significant differences for self-generated stimuli (stem). In particular, logβ was significantly lower in delusional depressed (*p* < 0.0001) and schizophrenic (*p* = 0.0353) patients compared to controls (Figure 1). 

Measures of observer sensitivity (d') and response criterion (β) for all three different sources are shown in Table 2. 

**Table 2:** Measures of observer sensitivity (d') and response criterion (β) for all three different sources.

		Mood disorder		Schizophrenia		Controls		
		Delusional (n = 21)	Non delusional (n = 23)	Delusional (n = 35)	Non delusional (n = 19)	(n = 50)	F	*p*
**Stem**								
	d'	2.68(0.83)	1.16(2.11)	2.55(0.66)	2.13(1.27)	3.00(0.69)	9.99	< 0.001
	log β	1.28(1.99)	2.47(0.72)	1.78(1.24)	2.59(0.68)	2.32(0.80)	5.33	< 0.001
**Picture**								
	d'	3.87(0.97)	4.03(0.79)	3.06(1.03)	3.20(1.57)	4.24(0.72)	9.11	< 0.001
	log β	−0.04(1.32)	0.42(1.19)	0.62(1.30)	1.19(1.33)	0.99(1.26)	3.36	0.03
**Heard**								
	d'	1.41(0.59)	1.78(0.71)	1.22(0.88)	1.34(0.54)	2.13(0.62)	10.79	< 0.001
	log β	1.51(0.79)	1.31(0.58)	1.32(1.01)	1.65(0.79)	1.76(0.86)	1.83	0.12

We also found differences between delusional and non-delusional patients: logβ was significantly lower for delusional depressed patients than for non-delusional ones (*p* < 0.0001) and for delusional schizophrenic patients than for non-delusional ones (*p* = 0.014). 

## 4. Discussion

Previously delusional depressed patients and currently delusional schizophrenic patients share a bias toward personalization and abnormally lax criteria in information processing of self-generated verbal material. These biases were absent in never-delusional depressed patients and in currently non-delusional schizophrenic patients, who had a similar performance to control subjects.

An abnormal process of reality testing of ongoing perception and reality monitoring of memories and beliefs, characterized by the use of inappropriately lax criteria in evaluating mental experiences, has been proposed as a neuropsychological correlate for development and maintenance of delusions [2,5].

Our observations in euthymic mood disordered and schizophrenic patients are in agreement with this hypothesis, and suggest that selective biases in reality monitoring are trait characteristics of patients who experience delusional symptoms. Following this line of reasoning, nuclear abnormalities of reality monitoring should be conceived as individual characteristics, which could lead to the development of delusions in the presence of affective or schizophrenic illness.

Despite a great amount of literature supporting this hypothesis some negative finding are present. Achim and Weiss report that, in schizophrenic patients, the associative memory deficit is not specific to self/other distinctions, but is rather a more global effect seen across testing conditions [36]. Externalizing bias represents a greater tendency to attribute negative as opposed to positive events to external causes; depressed patients tend to over attribute negative events to an internal causes and the schizophrenic patients do the opposite [27]. This could explain results obtained for the EB index.

## 5. Conclusions

The current article provides evidence of the presence of an abnormally lax criteria in information processing of self-generated verbal material for delusional patients (depressed and schizophrenic). This finding suggests that there may be a “psychotic dimension” that occurs in all of these syndromes and that should be considered in future classification systems [37].

Both affective disorder and schizophrenia are strongly familial. Some studies report an excess of mood disorders in relatives of schizophrenia probands. In addition, some studies report an excess of schizophrenia in relatives of mood disorders [38]. Familial and genetic factors may contribute to the risk of ‘‘psychosis in general’’ (schizophrenia spectrum or psychotic mood disorders), and some authors have suggested that the presence of schizophrenia in relatives could predispose people with affective disorders to have psychotic features [39]. Brain imaging studies have advanced our understanding of the neurobiology of schizophrenia and psychotic disorders. In many instances, brain abnormalities in psychotic and mood disorders appear to be on a spectrum, with the most marked changes in schizophrenia, followed by psychotic mood disorders, followed by nonpsychotic mood disorders [40].

Further research is needed to clarify these issues. In particular, future studies will define the continuity *vs.* discontinuity of the observed differences in respect with general population and other psychiatric conditions.

Dimensional and categorical approaches to psychoses have important limitations. However, technical and conceptual advances promise to continue to yield clinical and preclinical data that will improve psychiatric models.
